# AI-Based Myocardial Segmentation and Attenuation Mapping Improved Detection of Myocardial Ischemia and Infarction on Emergency CT Angiography

**DOI:** 10.3390/bioengineering13030355

**Published:** 2026-03-18

**Authors:** Martin Segeroth, Jan Vosshenrich, Hanns-Christian Breit, Helge Walter Anand Krebs-Fleischmann, Lorraine Abel, Markus Obmann, Shan Yang, Joshy Cyriac, Jakob Wasserthal, Ashraya Kumar Indrakanti, Michael Bach, Michael J. Zellweger, Alexander Sauter, Jens Bremerich, Philip Haaf, David Jean Winkel

**Affiliations:** 1 Department of Radiology and Nuclear Medicine, University Hospital Basel, 4031 Basel, Switzerlanddavidjean.winkel@usb.ch (D.J.W.); 2 Department of Cardiology, Cardiovascular Research Institute Basel, University Hospital Basel, 4031 Basel, Switzerland; 3 Department of Radiology and Nuclear Medicine, Stadtspital Zürich, 8063 Zürich, Switzerland; 4 Diagnostic and Interventional Radiology, Department of Radiology, Eberhard Karls University of Tübingen, 72076 Tübingen, Germany; 5 Nuclear Medicine and Clinical Molecular Imaging, Department of Radiology, Eberhard Karls University of Tübingen, 72016 Tübingen, Germany

**Keywords:** tomography, X-ray computed, myocardial infarction, acute coronary syndrome, deep learning

## Abstract

**Purpose:** To investigate whether an AI-based approach combining deep learning myocardial segmentation with attenuation-normalized myocardial mapping (colormaps) improves detection of myocardial ischemia and infarction on emergency ECG-gated CT angiography. **Materials and Methods:** In this retrospective study, 119 patients with acute chest pain who underwent ECG-gated CT angiography to exclude pulmonary embolism or acute aortic syndrome and invasive coronary angiography within 48 h were included. A deep learning model (nnU-Net) was used for automatic left-ventricular myocardial segmentation, serving as the basis for voxel-wise attenuation normalization to generate AI-based myocardial attenuation maps. Six readers with varying experience levels evaluated all cases for myocardial hypoattenuation in a multi-reader, multi-case design, with and without AI-generated attenuation maps. **Results:** AI-based myocardial attenuation mapping increased mean sensitivity for detection of myocardial ischemia or infarction by 12% [IQR 2–20%] compared with standard CT interpretation alone. Sensitivity improved by 15% [IQR 10–22%] in STEMI (ST-Elevation Myocardial Infarction) and 11% [IQR −1–18%] in NSTEMI (Non-STEMI) cases. The AI-assisted approach resulted in the correct reclassification of 11% of patients and improved inter-reader agreement, particularly among less experienced readers, demonstrating reduced reader dependency. **Conclusions:** AI-based myocardial segmentation and attenuation mapping enhance the detection of myocardial ischemia and infarction on emergency CT angiography and improve inter-reader agreement. This AI-assisted image processing approach provides clinically meaningful decision support in acute chest pain imaging workflows.

## 1. Introduction

Chest pain is the second most common reason for admission to the emergency department (ED), accounting for approximately 8 million patient visits annually [1,2]. As pointed out by the Chest Pain Guidelines from 2021 [3], there are multiple reasons for chest pain across all age groups. Among other etiologies, acute myocardial infarction, acute aortic syndrome (AAS) and pulmonary embolism (PE) are some examples of etiologies often requiring immediate action. Thus, the differential diagnosis of chest pain is complex [4]. Of the 8 million patients that present to the ED with chest pain, only 10% to 25% are ultimately diagnosed with acute coronary syndrome (ACS), and thereof only one-third with acute myocardial infarction (AMI) [1,5,6].

Patients with a missed diagnosis of ACS tend to be younger, more frequently females, have an atypical presentation for ACS or a non-diagnostic electrocardiogram (ECG) [7,8]. In such patients, due to the nonspecific presentation, other differential diagnoses, such as AAS or PE, are excluded using ECG-gated CT angiographies in the emergency setting [3]. This may lead to a possible delay of an atypical ACS. To account for this problem, a dedicated coronary CTA in addition to an ECG-gated CTA of the chest and abdomen can be performed to assess AAS, PE and ACS with one single CT examination, sometimes referred to as “triple-rule out, TRO” [4,9,10,11,12].

This technique relies on the visualization of the vessel lumen using a contrast agent to detect or exclude a stenosis or occlusion of a coronary vessel for the diagnosis of an ACS.

The myocardium, though, has another well-known feature that allows to diagnose myocardial abnormalities in the absence of a diagnostic coronary CTA: it is hypoattenuating in cases of myocardial ischemia and acute or chronic myocardial infarction [12,13,14].

In this study, we aimed to develop and validate myocardial colormaps in a patient population with acute chest pain who received ECG-gated CT angiographies to exclude pulmonary embolism and acute aortic syndrome, and to systematically investigate the added value of myocardial colormaps in ECG-gated CTAs. We sought to assess the effects of overlaying myocardial colormaps using a multi-reader, multi-case (MRMC) study. Our hypothesis was that complementary colormaps would lead to an improved detection of MI and higher inter-reader agreement of myocardial abnormalities, such as ischemia or infarction, compared to a scenario without colormaps.

## 2. Materials and Methods

### 2.1. Patients

The Ethics Committee of Northwestern and Central Switzerland (EKNZ—Ethikkommission Nordwest-und Zentralschweiz) approved this study (BASEC-2021-01986). We retrospectively analyzed patients meeting all the following inclusion criteria: (a) patients who underwent an ECG-gated computed tomography angiography examination due to acute chest pain and suspicion for either pulmonary embolism (PE) or acute aortic syndrome (AAS), while ACS could not be ruled out at presentation, (b) invasive coronary angiogram not later than 48 h after the CT examination, and (c) age ≥ 18 years at the time of examination. Patients were included from January 2016 to October 2021. Using all clinical information extracted from the electronic health record (electrocardiography and Troponin T/I), patients were adjudicated to the presence of ST-elevated myocardial infarction (STEMI), non-ST-elevated myocardial infarction (NSTEMI) or no evidence of ACS.

### 2.2. CT Examinations

Patients underwent ECG-triggered, arterial phase CT of the chest and abdomen on one of 4 different CT scanners (Siemens Healthineers, Erlangen, Germany, SOMATOM Definition Flash/Edge/AS+/Force). Intravenous contrast (100–130 mL Ultravist 370, Bayer, Leverkusen, Germany or Iopamiro 370, Bracco, Milan, Italy) was applied, and the scan was then initiated using bolus triggering. Patients did not receive any specific medication before the examination, such as beta-blockers or Glyceryltrinitrate. Automatic tube current modulation was used with a reference mAs between 181 and 320 and a tube voltage of 80–120 kVp, depending on patient size. Images were reconstructed in mid-to-late diastole (best diastolic phase), which was automatically determined by the scanner based on the patient’s ECG signal with a slice thickness of 0.6 mm and a slice increment of 0.3 mm using a 512 × 512 matrix, a soft tissue kernel (I26f or Bv40d) with iterative reconstruction (ADMIRE 3). The reconstructed field of view of the reconstruction was adjusted to include the complete heart. The in-plane pixel size and number of reconstructed slices varied depending on the individually selected field of view and scan range. A detailed list of scanning parameters can be found in the Supplementary Materials (Supplementary Materials S1). Each examination was pre-checked for sufficient image quality.

### 2.3. Radiology Reports

Reports were retrieved from the RIS (Radiology Information System) and were written in clinical routine with a peer review. The report is built of modules (e.g., CHEST and ABDOMEN). Each module has specific sub-items. Relevant for this study were the sub-items “Aorta”, “Pulmonary Vasculature” and “Heart”. Here, the reader is asked to insert specific information, including a description of the proximal coronary arteries and the myocardium. For the final study population, CT reports were reviewed for the presence/absence of vessel stenosis/occlusion, hypodense myocardium, and myocardial ischemia/infarction.

### 2.4. Invasive Coronary Angiography

Invasive coronary angiography was performed according to the standard Judkin’s technique. Whenever possible, radial artery access was used. In a minority of patients, femoral artery access was used. The invasive coronary angiography reports at our institution provide a table-based, structured assessment of the coronary anatomy per segment according to established reporting systems [15].

### 2.5. Myocardial Segmentation and Color Mapping

Myocardial segmentation resulted from a pre-trained nnU-Net (trained on data from MM-WHS: Multi-Modality Whole Heart Segmentation [16]) for automatic segmentation of the cardiac structures, including left and right ventricle, the myocardium, both atria, the aorta and the pulmonary trunk. Hereafter, the segmentation of the myocardium was manually revised independently by two readers, with an experience of six and two years, respectively. The difference between the manual and automated segmentation was evaluated using the Dice score.

The manually refined segmentation of the myocardium was used to calculate the colormaps of the myocardium. These colormaps represent the voxel-wise deviation from the mean myocardial attenuation, normalized by the intra-myocardial attenuation variance. First, the mean attenuation of the myocardium is voxel-wise subtracted from each attenuation value of the myocardium. Second, the resulting values are divided by the variance of the 10th to 90th quantile attenuation values. The 10th to 90th quantile was chosen to account for single voxel outliers within the myocardium.

Since attenuation values were normalized to the individual mean myocardial attenuation and intra-myocardial variance, global differences in absolute attenuation (e.g., related to tube voltage variation) do not influence the resulting relative deviation colormaps.

### 2.6. MRMC Study Design

Our MRMC study was performed according to established standards [17]. We randomly assigned the six readers (2 junior residents: <1 year of experience in cardiothoracic imaging, 2 experienced residents: >4 years of experience in cardiothoracic without dedicated sub-specialization, and 2 experts: European Association of Cardiovascular Imaging (EACVI) Level 3) to two reader groups with a representative of each experience level in each group (group 1: readers 1–3, group 2: readers 4–6). All readers received training to get familiar with the reading platform 2 weeks before the actual start of the reading sessions. The 119 cases were divided into two case groups (group A: 1–60, group B: 61–119). Subsequently, we assigned the case group A to the reader group I with overlaying myocardial colormaps and to the reader group II without overlaying myocardial colormaps. Similarly, the case group B was assigned to the reader group I without overlaying myocardial colormaps and to the reader group II with overlaying myocardial colormaps. After an interval of at least two weeks, the assignments were crossed. With this set-up, every reader read every study both with and without overlaying myocardial colormaps, resulting in 1428 readings (6 readers × 119 cases × 2). All readers were blinded to these results.

### 2.7. Reading

Readings were performed on NORA (https://www.nora-imaging.com/ (accessed on 11 March 2026)), a web-based framework for medical image analysis [18]. NORA allows visualizing, organizing, processing and sharing data in a highly configurable way through a high-level web interface accessible from any web browser. Specific accounts with integrated reading sessions (with and without overlaying colormaps) were created for each reader on the NORA imaging platform and were distributed to the readers. For both scenarios, with and without colormaps, readers were instructed to evaluate the presence of a hypodense myocardium with the categories “Yes” and “No”, whereas the readers were allowed to evaluate the coronary vessels to pinpoint the hypodense myocardium. Readers had the possibility to adjust the image plane to specific needs (e.g., to create short-axis views). Next, readers were asked to assign a vessel territory to the area of hypodense myocardium: “right coronary artery (RCA)”, “left anterior descending artery (LAD)” and “ramus circumflexus (CX)”. Thus, readers were allowed to evaluate the coronary arteries; however, without assessing them in a systematic way, as this was not the scope of the study. Finally, the readers were asked to rate their certainty of the hypodense myocardium: “0–10%: definitively no”, “10–40%: probably no”, “40–60%: intermediate”, “60–90%: probably yes” and “90–100%: definitively yes”. In the session with supportive colormaps, the readers also had the normal CT without an overlying colormap. NORA automatically measured the time for each case and each reader.

### 2.8. Statistical Analysis

Continuous variables were reported as means +/− SD when normally distributed and as medians (interquartile range [IQR]) when otherwise. Normality was verified by a visual approach using frequency histograms and quantification using the Kolmogorov–Smirnov test. According to [17], sensitivity, specificity, accuracy, and positive and negative predictive values are given for every reader in the scenario with all cases with supportive colormaps and without colormaps. Since the readers were not able to rate for STEMI and NSTEMI but only for hypodense myocardium with STEMI and NSTEMI, only sensitivity and positive predictive value were reported. Comparison of sensitivity and specificity was performed using McNemar’s test. Bar charts were used for the visualization of distributions of detection rates. Inter-reader agreement was described by Cohen’s kappa. Comparisons between the scenario with and without colormaps were conducted using the binary net reclassification index (hypoattenuation vs. no hypoattenuation). The net reclassification index (NRI) is an index that attempts to quantify how well a new model or test reclassifies subjects as compared to an old model or test [19]. The NRI can be described with an interval between −1 and 1, where 1 means that the new model or test is better than the old model or test. On the other hand, −1 indicates that the new model or test is worse compared to the old model or test. For the interpretation of the NRI, the value can be expressed as a percentage of all included patients that were correctly reclassified [20]. All statistical analyses were performed using Python 3.8.8 (Python Software Foundation, Wilmington, DE, USA).

## 3. Results

**Figure 1:**
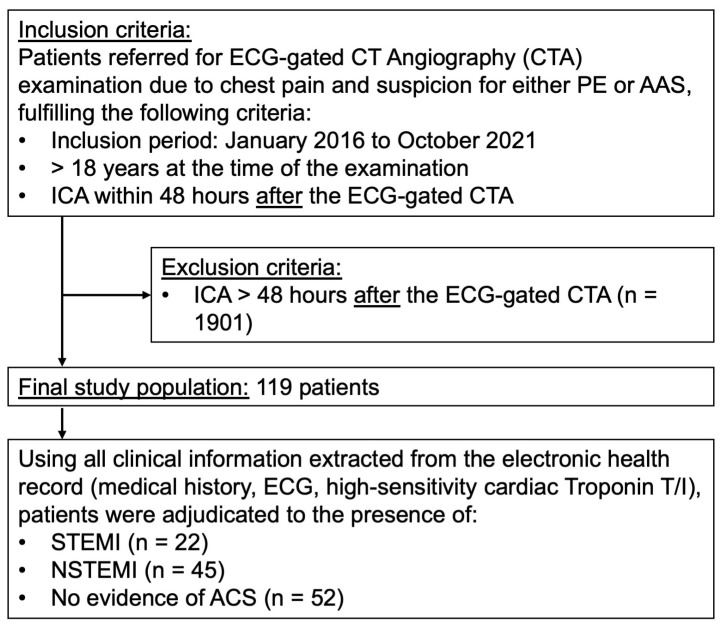
Flowchart outlining the selection of the final study population with utilized inclusion and exclusion criteria within the defined observation window. CT = computed tomography, PE = pulmonary embolism, AAS = acute aortic syndrome, ICA = invasive coronary angiography, ECG = electrocardiography, STEMI = ST-elevation myocardial infarction, NSTEMI = non ST-elevation myocardial infarction.

From January 2016 to October 2021, a total of 2020 emergency ECG-gated CT angiography examinations to rule out AAS or PE were performed. All patients were older than 18 years at the time of the examination. Of these 2020 examinations, 149 patients (7%, 149/2020) underwent an invasive coronary angiography (ICA) within a range of 0–1803 days after the CT examination. A total number of 119 patients had an ICA within 48 h after the CT examination, thus meeting the inclusion criteria and qualifying for the final study population (see Figure 1). In the final study population, the mean age was 66.27 ± 13.57 years, and 86 of 119 patients (72%) were male.

Regarding the segmentation accuracy of the myocardium between the automated and manual segmentations, the average Dice score of the model was 0.973 for reader one and 0.985 for reader two.

A total of 67 of 119 included patients (56%) had a myocardial infarction or ischemia (STEMI: 22, NSTEMI: 45).

### 3.1. Reports: Myocardial Infarction

A MI was stated in seven (6%, 7/119) of the radiology reports. All of them had a MI (four STEMIs and three NSTEMIs), yielding a sensitivity of 10% (7/67), specificity and positive predictive value of 100% and a negative predictive value of 46%. In seven (6%) out of all radiology reports, a hypodense myocardium was described. All of them had a MI (four STEMIs and three NSTEMIs), yielding a sensitivity of 10% (7/67), but in only 4/7 patients, the observation of a hypodense myocardium was interpreted as a MI. In 28 patients, a relevant stenosis or occlusion was observed, whereas 26/28 had a MI (17 STEMIs and nine NSTEMIs), yielding a sensitivity of 39% (26/67), but in only 4/28 of these patients, this resulted in the impression of a MI.

We additionally evaluated a broader definition of reported myocardial infarction. If coronary artery vessel stenosis or occlusion OR hypodense myocardium OR direct statement of a MI in either the findings or impression section of the radiology report had been considered indicative of MI, sensitivity would have been 43%, specificity 94%, positive predictive value 91% and negative predictive value 56%.

### 3.2. Reading: Myocardial Infarction Detection

**Figure 2:**
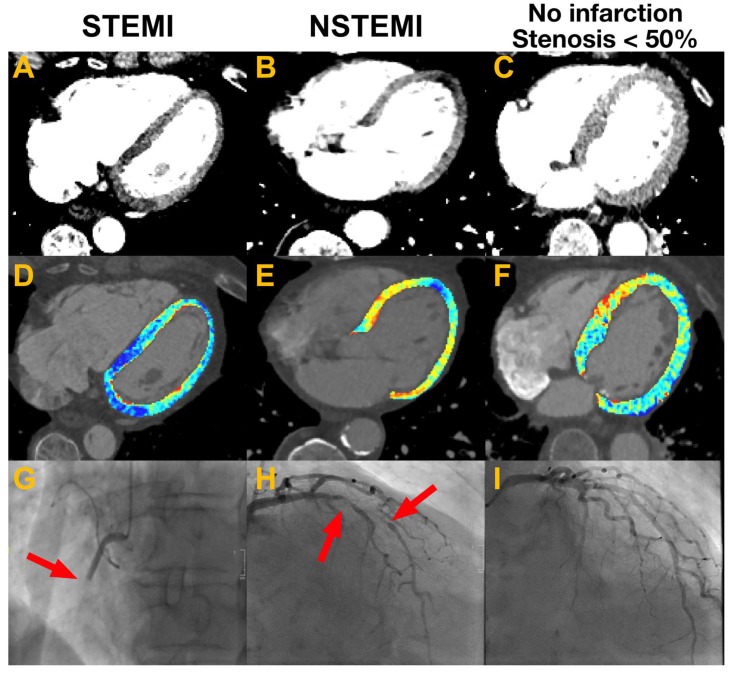
Figure showing the heart from a CT examination without (first row; (**A**–**C**)) and with (second row; (**D**–**F**)) overlying colormaps for cases with a STEMI, NSTEMI and no myocardial infarction and vessel stenosis < 50%. Voxels with a negative deviation from the mean of the myocardium are shown in blue; voxels with a positive deviation are shown in red. The third row shows the corresponding invasive coronary angiographies (ICA) with a total proximal occlusion of the RCA for the STEMI (**G**), a high-grade stenosis of the LAD for the NSTEMI (**H**) and no hemodynamically relevant stenosis in the case with no infarction (**I**). Arrows indicate the location of vessel occlusion or high-grade stenosis in the angiographic images. The case with the STEMI shows a transmural hypodensity of the inferior wall (**A**,**D**). The case with the NSTEMI demonstrates a small subendocardial hypodensity (**B**,**E**). In the case with no relevant vessel stenosis, no hypodense myocardium was described (**C**,**F**).

Figure 2 visualizes readings with and without colormaps and correlated findings from the ICA for a representative set of cases with STEMI, NSTEMI and no coronary vessel occlusion > 50%. Table 1 summarizes the relevant metrics of diagnostic accuracy. The use of supportive colormaps increased sensitivity for myocardial abnormalities on average by 12% (from 34% to 46%) with a significant increase in three readers. Specificity decreased by 8% (from 83% to 75%), significantly in two readers. The overall accuracy increased by 3% (from 55% to 58%). The positive predictive value decreased slightly by 2% (from 74% to 72%), and the negative predictive value slightly increased by 3% (from 49% to 52%). According to the average net reclassification index (NRI), 11% of patients were correctly reclassified when comparing the scenario with versus without colormaps. An exemplary net reclassification index calculation is shown in Table 2 for reader 4.

**Figure 3:**
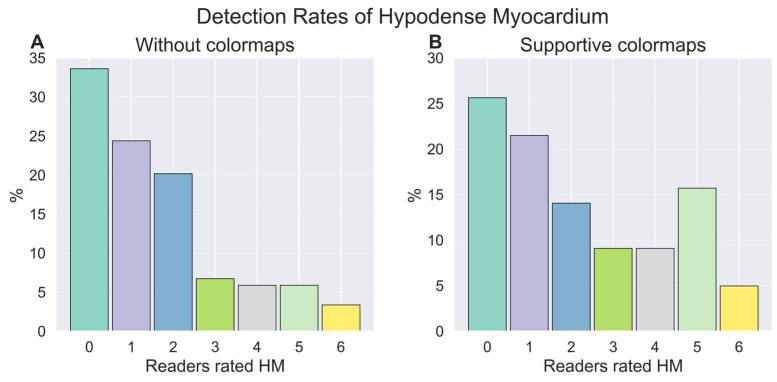
(**A**) Percentage of readers that rated hypodense myocardium (HM) in each case without colormaps. (**B**) Percentage of readers that rated hypodense myocardium in each case with supportive colormaps. The colors of the bars are used only for visual distinction and do not represent additional categories.

**Figure 4:**
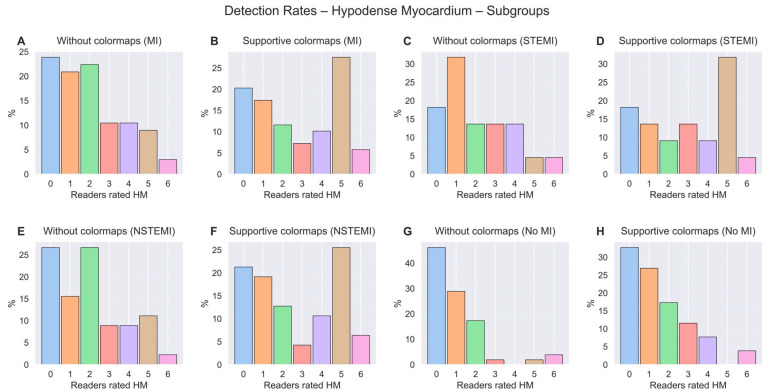
(**A**) Percentage of readers that rated hypodense myocardium (HM) in each case for the subgroup of patients with a myocardial infarction without colormaps, and (**B**) with supportive colormaps. (**C**) Percentage of readers that rated hypodense myocardium (HM) in each case for the subgroup of patients with a STEMI without colormaps, and (**D**) with supportive colormaps. (**E**) Percentage of readers that rated hypodense myocardium (HM) in each case for the subgroup of patients with a NSTEMI without colormaps, and (**F**) with supportive colormaps. (**G**) Percentage of readers that rated hypodense myocardium (HM) in each case for the subgroup of patients without a myocardial infarction without colormaps, and (**H**) with supportive colormaps. The colors of the bars are used only for visual distinction and do not represent additional categories.

Regarding reader experience, colormaps showed the highest increase in sensitivity for experienced residents (19%), followed by experts (15%) and junior residents (4%). Of note, expert reader 6 had almost no changes between with and without colormaps (Table 1). Figure 3A,B demonstrate that, with supportive colormaps, more readers detected a hypodense myocardium compared to a scenario without colormaps in all cases. Figure 4A,B show the same effect when myocardial infarction is present.

### 3.3. Sub-Classification of STEMI vs. NSTEMI and Vessel Territory

Table 3 summarizes the relevant metrics for the sub-classification. For STEMI cases, sensitivity increased on average by 15% (from 34% to 49%) and, for NSTEMI cases, by 11% (from 33% to 44%). Regarding reader experience, colormaps showed a higher increase in sensitivity for experienced residents, followed by experts and junior residents (STEMI: 23% vs. 11% vs. 11% increase; NSTEMI: 17% vs. 16% vs. 0% increase). Again, with reader 6, almost no changes with supportive colormaps were observed. Figure 4C,D demonstrate that, in the case of a STEMI, more readers detected a hypodense myocardium with supportive colormaps compared to a scenario without colormaps. This was also found in the case of a NSTEMI (Figure 4E,F).

Regarding the affected vessel territory, only a strong positive effect in terms of sensitivity was observed for the CX with an increase of 10% (33% vs. 23%) (Table 4). For the other vessel areas, no relevant improvement was detected.

### 3.4. Inter-Reader Agreement

**Figure 5:**
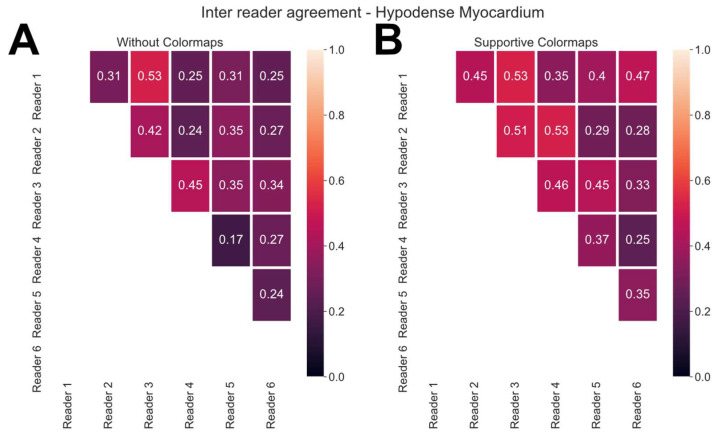
Inter-reader agreement for hypodense myocardium with on average lower kappa values without colormaps (**A**) and on average higher kappa values with supportive colormaps (**B**). On average, the use of colormaps allowed less experienced readers to achieve a higher agreement with expert readers concerning the detection of a hypodense myocardium (**A**,**B**).

Regarding the detection of a hypodense myocardium, readers 1 and 2 had a higher agreement with more experienced readers if they had supportive colormaps. This also applied to readers 3 and 4 (Figure 5A,B, Supplementary Materials S2). Although with readers 1 and 2 as junior residents, readers 3 and 4 as experienced residents and readers 5 and 6 as experts with a similar level of experience, their kappa between each other did increase as well if they had supportive colormaps (Figure 5A,B). Regarding the reader’s probability of a hypodense myocardium, similar effects were recorded (Supplementary Materials S2).

### 3.5. Time

In general, there was no difference in the time the readers needed on average for one reading between without colormaps, 27 s (IQR 16 to 43 s), and with colormaps, 25 s (IQR 17 to 42 s; *p* = 0.49). This also accounts for readers 1 and 2. Readers 3 and 6 were significantly faster without the colormaps (19 s (IQR 14 to 26 s) vs. 23 s (IQR 18 to 38 s); *p* < 0.0001; 28 s (IQR 22 to 40 s) vs. 34 s (IQR 23 to 49 s); *p* < 0.0001). In contrast, readers 4 and 5 were significantly faster with supportive colormaps (16 s (IQR 12 to 24 s) vs. 22 s (IQR 14 to 29 s); *p* = 0.002; (44 s (IQR 27 to 75 s) vs. 67 s (IQR 54 to 89 s); *p* < 0.0001).

## 4. Discussion

We report five major findings:

- First, in only a fraction of the peer-reviewed radiology reports written in clinical routine (7/119), the findings were clearly interpreted as a myocardial ischemia/infarction.
- Second, the use of supportive colormaps led to an improved detection of myocardial hypodensities (11% of the study population were correctly reclassified) compared to a scenario without colormaps, with a significant increase in sensitivity for three readers.
- Third, the use of additional colormaps allowed the readers to increase their sensitivity in cases with STEMI by 15% (49% vs. 34%) and in cases with NSTEMIs by 11% (44% vs. 33%) compared to a reading scenario without colormaps.
- Fourth, particularly senior residents, who are more frequently exposed to the emergency setting, benefited most from the colormaps, with significant increases in sensitivity for both readers.
- Fifth, the use of colormaps allowed less experienced readers to achieve a higher agreement with expert readers concerning the detection of a hypodense myocardium.

This study systematically evaluated the effect of supportive colormaps of the left-ventricular myocardium on the diagnostic accuracy and inter-reader agreement in the setting of a multi-reader, multi-case study for STEMI and NSTEMI.

Computed tomography-based approaches to assess myocardial infarction or ischemia were already developed in the late 1970s. Adams, D.F. et al. used a single-slice spiral CT to demonstrate areas of lower attenuation within the normal enhanced myocardium in cases of myocardial infarction [21]. This technique has been refined to myocardial perfusion imaging [14], either with single-phase [22] or multi-phase acquisition [23] schemes, showing promising results. However, these techniques rely on the comparison of stress/rest images. As our CT datasets were acquired in the emergency setting to rule out PE and AAS, speed was one prerequisite in the diagnostic workup. Thus, myocardial perfusion imaging could not have been used.

Nevertheless, hypoattenuation of the myocardium in cases of myocardial ischemia and acute or chronic myocardial infarction is a known feature [12,13,14]. Previous studies in the past have shown quantifiable attenuation differences of up to 80 Hounsfield units (HU) [11] in cases of acute infarction. Confounding factors, however, may be the severity of coronary artery disease, the extent of collateralization, and the time between the onset of symptoms and the CT examination. This issue might be solved by means of overlaying colormaps for better visualization of otherwise grayscale encoded HU values. The added value of such colormaps has been used in a variety of body regions, such as the liver [24] or brain [25], showing promising results.

In contrast to this approach, in recent years, the so-called “triple-rule-out” approach has gained increasing attention, as the aorta, coronary circulation and pulmonary arteries can be evaluated simultaneously within one scanning procedure [4,9,10,11]. Advantages of a TRO approach are a reduced need for additional diagnostic testing in >75% of patients with a low to intermediate pre-test probability [26] and a generally high negative predictive value of a coronary CTA [27]. However, a TRO approach is not commonly implemented in clinical routine, with only a minority of radiology practices performing TRO examinations [28]. Potential reasons are inconsistent image quality, technical difficulties, the necessity of pre-medication before the exam or compromised visualization of the coronary arteries using the TRO approach; yet, this technique relies on the visualization of the vessel lumen using a contrast agent to detect or exclude a stenosis or occlusion of a coronary vessel for the diagnosis of an ACS. Russo et al. [29] demonstrated that radiologists without cardiovascular sub-specialization were able to exclude the presence of an obstructive stenosis in a triple rule-out CT setting. Our results support this finding, since partial/obstructive vessel occlusion was initially described in the radiology report in most STEMI cases in our patient cohort. Nevertheless, this observation did not lead to the impression of a myocardial infarction in the peer-reviewed radiology reports, indicating there might be an insecurity regarding this diagnosis. This likely reflects the limited sensitivity of routine visual myocardial assessment in CT examinations primarily performed to exclude pulmonary embolism or acute aortic syndrome.

To support the confidence in a diagnosis of MI or in cases of a hampered evaluation of the lumen of the coronary artery, for example, due to strong calcification [30], the attenuation of the myocardium itself may serve as a diagnostic marker of an upstream vessel occlusion. Especially in a scenario in which patients do not receive specific pre-medication such as beta-blockers or Glyceryltrinitrat to lower the heart rate and dilate the coronary arteries, the evaluation of the myocardium may serve as a very helpful diagnostic marker.

Mahnken et al. [31] demonstrated in 15 patients that hypodense segments of the myocardium with the 17-segment model can be identified with a higher accuracy and a higher inter-reader agreement using dedicated post-processing algorithms that highlight hypodense myocardium as a surrogate for myocardial infarction. We confirm these findings by observing a higher inter-reader agreement with supportive colormaps.

Our colormaps allowed senior residents, who are often exposed in an emergency setting and are the first to communicate findings to referring physicians, to achieve a sensitivity for myocardial ischemia/infarction of up to 52%, with a significant increase in sensitivity with colormaps for both readers compared to a scenario without. This might increase the confidence of a radiologist in the emergency setting to interpret imaging evidence as a pathological finding (e.g., the presence of a MI) and might thus lead to faster treatment/dedicated diagnostic workup. Colormaps showed no significant effect on the reading performance of less experienced readers when all cases are considered. However, colormaps helped less experienced readers to increase their sensitivity in the detection of STEMI cases. Regarding expert readers, the results are mixed, as one reader showed a significant benefit in the detection of hypodense myocardium using colormaps, while another reader showed an already high performance without colormaps and no increase with the use of colormaps. Overall, the detection of a MI increased using additional colormaps, as indicated by the positive NRI.

It must be mentioned that specificity decreased significantly in two readers. This could imply that in a scenario without colormaps, fewer hypodensities are reported, resulting in an artificially high specificity (reader 5, Table 1), likely reflecting a conservative diagnostic threshold prioritizing specificity over sensitivity. Accuracy was on average 3% higher with supportive colormaps; however, this difference was small and not statistically significant. The results of our study show that the use of myocardial colormaps in patients who received an emergency ECG-gated CTA due to suspicion of PE or AAS and possible ACS leads to a diagnostic advantage versus a scenario without colormaps.

By providing additional colormaps routinely in the future, awareness to evaluate the myocardium and confidence to diagnose myocardial abnormalities may be increased.

Limitations of this study were: First, as a retrospective study, we cannot quantify exactly the clinical benefit of the colormaps for further diagnostics and therapy. Further prospective studies need to be conducted to evaluate the actual impact of the technique. Second, the sample size is limited to patients who received an ECG-gated CTA and ICA within 48 h. This may have led to a selection bias.

In conclusion, supportive colormaps of the myocardium led to a higher overall sensitivity to detect myocardial abnormalities such as infarction or ischemia, both in STEMI and NSTEMI cases, and further led to a higher inter-reader agreement between less and more experienced readers. Thus, the colormaps may help to guide and accelerate further diagnostics, including invasive coronary angiography.

## Figures and Tables

**Table 1:** Metrics of diagnostic accuracy per readers and experience level with and without supporting colormaps. wc = without colormap; sc = supportive colormap.

Experience Level	Reader	Sensitivity	Specificity	Accuracy	Positive Predictive Value	Negative Predictive Value	McNemar-Test	Net Reclassification Index
Junior resident	Reader 1 wc	46.3%	92.3%	66.4%	88.6%	57.1%	Sensitivity: *p* = 1 Specificity: *p* = 0.22	−6%
	Reader 1 sc	46.4%	84.6%	62.8%	80.0%	54.3%		
Junior resident	Reader 2 wc	55.2%	55.8%	55.5%	61.7%	49.2%	Sensitivity: *p* = 0.30 Specificity: *p* = 1	8%
	Reader 2 sc	62.7%	55.8%	59.7%	64.6%	53.7%		
Experienced resident	Reader 3 wc	28.4%	94.2%	57.1%	86.4%	50.5%	Sensitivity: *p* = 0.006 Specificity: *p* = 0.016	7%
	Reader 3 sc	43.3%	80.8%	59.7%	74.4%	52.5%		
Experienced resident	Reader 4 wc	29.9%	76.9%	50.4%	62.5%	46.0%	Sensitivity: *p* = 0.008 Specificity: *p* = 0.12	21%
	Reader 4 sc	52.2%	63.5%	57.1%	64.8%	50.8%		
Expert	Reader 5 wc	13.4%	92.3%	47.9%	69.2%	45.3%	Sensitivity: *p* < 0.0001 Specificity: *p* = 0.022	35%
	Reader 5 sc	44.9%	75.0%	57.8%	70.5%	50.6%		
Expert	Reader 6 wc	28.4%	86.5%	53.8%	73.1%	48.4%	Sensitivity: *p* = 0.80 Specificity: *p* = 0.69	1%
	Reader 6 sc	26.1%	90.4%	53.7%	78.3%	48.0%		
Average	Average wc	33.6%	83.0%	55.1%	73.5%	49.4%		11%
	Average sc	45.9%	75.0%	58.4%	72.1%	51.65%		

**Table 2:** Exemplary binary net reclassification index. Bold cases were correctly classified in both reading with CT only and CT with supportive colormaps; light blue cases were incorrectly classified by both; green cases were correctly reclassified using CT colormaps; and red cases were incorrectly reclassified using CT colormaps.

Case		CT		Total, Split	Total
Non Case		Abnormal	Normal		
CT Colormaps	Abnormal	**13**	22	35	71
		25	11	36	
	Normal	7	8	15	48
		4	**29**	33	
Total, split		20	30		
		29	40		
Total		49	70		
NRI	0.21				

**Table 3:** Metrics of diagnostic accuracy per reader and experience level divided for STEMI and NSTEMI cases. Values in parentheses demonstrate the metrics without colormaps.

		Sensitivity	Positive Predictive Value
STEMI	Reader 1	63.6% (50.0%)	35.0% (31.4%)
	Reader 2	63.6% (54.5%)	21.5% (20.0%)
	Reader 3	50% (31.8%)	28.2% (31.8%)
	Reader 4	59.1% (31.8%)	24.1% (21.9%)
	Reader 5	36.4% (13.6%)	18.2% (18.2%)
	Reader 6	22.7% (22.7%)	21.7% (19.2%)
	Average	49.2% (34.1%)	24.8% (23.8%)
NSTEMI	Reader 1	38.3% (44.4%)	45.0% (57.1%)
	Reader 2	62.2% (55.6%)	43.1% (41.7%)
	Reader 3	40.0% (26.7%)	46.2% (54.5%)
	Reader 4	48.9% (28.9%)	40.7% (40.6%)
	Reader 5	48.9% (13.3%)	52.3% (46.2%)
	Reader 6	27.7% (31.1%)	56.5% (53.8%)
	Average	44.3% (33.3%)	47.3% (49%)

**Table 4:** Metrics of diagnostic accuracy pooled for all readers per coronary vessel territory. Values in parentheses demonstrate the metrics without colormaps. LAD = Left anterior descending; CX = Ramus circumflexus; RCA = Right coronary artery.

		Sensitivity	Specificity	Accuracy	Positive Predictive Value	Negative Predictive Value
LAD	Average	35.0% (34.2%)	73.9% (77.5%)	54.3% (56.0%)	63.2% (62.4%)	52.8% (54.7%)
CX	Average	32.5% (22.6%)	77.2% (90.2%)	62.4% (68.1%)	49.6% (61.7%)	69.7% (70.6%)
RCA	Average	11.1% (9.1%)	95.4% (94.4%)	66.1% (64.3%)	63.7% (38.8%)	66.9% (65.6%)

## Data Availability

Data generated or analyzed during the study are available from the corresponding author by request. The data are not publicly available due to ethical and institutional restrictions related to patient confidentiality.

## Supplementary Materials

The following supporting information can be downloaded at: https://www.mdpi.com/article/10.3390/bioengineering13030355/s1, Supplementary Materials S1: CT Examination Scan Parameters. Supplementary Materials S2: Inter-Reader Agreement–Probability for Hypodense Myocardium.

## Abbreviations

ACS	acute coronary syndrome
AAS	acute aortic syndrome
ED	emergency department
PE	pulmonary embolism
TRO	triple-rule out
ICA	invasive coronary angiography
STEMI	ST-elevation myocardial infarction
NSTEMI	non-ST-elevation myocardial infarction
