# Supplementary material for: AI-Based Myocardial Segmentation and Attenuation Mapping Improved Detection of Myocardial Ischemia and Infarction on Emergency CT Angiography
Source: Bioengineering (Basel). 2026 Mar 18;13(3):355. doi: 10.3390/bioengineering13030355 (PMC13024399; doi:10.3390/bioengineering13030355)
Supplement: Supplementary file 1 [file bioengineering-13-00355-s001.zip › bioengineering-4138514-supplementary.pdf]

SUPPLEMENTARY MATERIALS S1: CT Examination Scan Parameters

Scanner Model	Slice Thickness (mm)	Number of Examinations	Increment (mm)	Kernel	Iterative Reconstruction Algorithm	kVp	Reference mAs
AS+	0.6	85	0.3	i26f	ADIMRE 3	120	181/200
Edge	0.6	4	0.3	i26f	ADIMRE 3	120	181
Force	0.6	18	0.3	Bv40d	ADIMRE 3	100	282/250
Flash	0.6	12	0.3	i26f	ADIMIRE 3	80	320

SUPPLEMENTARY MATERIALS S2: Inter-Reader Agreement – Probability for Hypodense Myocardium

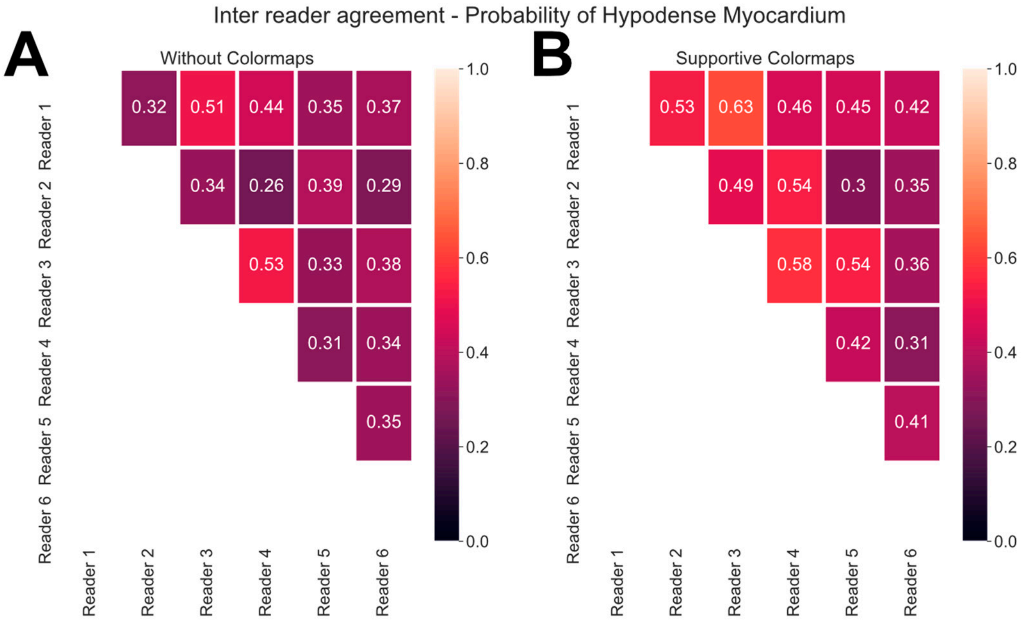

Inter-reader agreement for probability of hypodense myocardium with on average lower kappa values without colormaps (A) and on average higher kappa values with supportive colormaps (B). On average, the use of colormaps allowed less experienced readers to achieve a higher agreement with expert readers concerning the probability of a hypodense myocardium (A, B).
